# Enhanced tomato disease resistance primed by arbuscular mycorrhizal fungus

**DOI:** 10.3389/fpls.2015.00786

**Published:** 2015-09-28

**Authors:** Yuanyuan Song, Dongmei Chen, Kai Lu, Zhongxiang Sun, Rensen Zeng

**Affiliations:** ^1^College of Life Sciences, Fujian Agriculture and Forestry University, FuzhouChina; ^2^State Key Laboratory of Conservation and Utilization of Subtropical Agro-Bioresources, College of Agriculture, South China Agricultural University, GuangzhouChina

**Keywords:** defense priming, arbuscular mycorrhizal fungus, induced disease resistance, systemic defense responses, jasmonate pathway, tomato

## Abstract

Roots of most terrestrial plants form symbiotic associations (mycorrhiza) with soil- borne arbuscular mycorrhizal fungi (AMF). Many studies show that mycorrhizal colonization enhances plant resistance against pathogenic fungi. However, the mechanism of mycorrhiza-induced disease resistance remains equivocal. In this study, we found that mycorrhizal inoculation with AMF *Funneliformis mosseae* significantly alleviated tomato (*Solanum lycopersicum* Mill.) early blight disease caused by *Alternaria solani* Sorauer. AMF pre-inoculation led to significant increases in activities of β-1,3-glucanase, chitinase, phenylalanine ammonia-lyase (PAL) and lipoxygenase (LOX) in tomato leaves upon pathogen inoculation. Mycorrhizal inoculation alone did not influence the transcripts of most genes tested. However, pathogen attack on AMF-inoculated plants provoked strong defense responses of three genes encoding pathogenesis-related proteins, *PR1*, *PR2*, and *PR3*, as well as defense-related genes *LOX*, *AOC*, and *PAL*, in tomato leaves. The induction of defense responses in AMF pre-inoculated plants was much higher and more rapid than that in un-inoculated plants in present of pathogen infection. Three tomato genotypes: a Castlemart wild-type (WT) plant, a jasmonate (JA) biosynthesis mutant (*spr2*), and a prosystemin-overexpressing *35S::PS* plant were used to examine the role of the JA signaling pathway in AMF-primed disease defense. Pathogen infection on mycorrhizal *35S::PS* plants led to higher induction of defense-related genes and enzymes relative to WT plants. However, pathogen infection did not induce these genes and enzymes in mycorrhizal *spr2* mutant plants. Bioassays showed that *35S::PS* plants were more resistant and *spr2* plants were more susceptible to early blight compared with WT plants. Our finding indicates that mycorrhizal colonization enhances tomato resistance to early blight by priming systemic defense response, and the JA signaling pathway is essential for mycorrhiza-primed disease resistance.

## Introduction

In response to various abiotic stresses and biotic attack by herbivorous insects or pathogens, plants have evolved an array of sophisticated strategies to protect themselves against these agents. One strategy is the ability of plant root systems to form mycorrhizal associations, which are mutualistic and reciprocally beneficial symbiotic relationships between plant roots and some specific soil-borne fungi. Mycorrhizal fungi are the most important symbionts for the majority of plant species in terrestrial ecosystems (Smith and Read, 2008). It has been estimated that over 90% of land plants form arbuscular mycorrhizas (AM) with fungi belonging to the phylum Glomeromycota (Brundrett, 2002). The presence of widespread extra radical mycelium networks of mycorrhizal fungi in soils helps plants to acquire nutrients and water in soils which plant roots can not reach. Mycorrhizal associations facilitated the plant colonization on land (Redecker et al., 2000). Mycorrhizas also influence plant physiology (Smith et al., 2010) and soil structure (Rillig and Mummey, 2006; Fellbaum et al., 2012), as well as a series of important ecosystem processes, including plant diversity, nutrient cycling, and ecosystem productivity (van der Heijden et al., 1998; Vogelsang et al., 2006; Cheng et al., 2012).

Numerous studies have proven that arbuscular mycorrhiza fungi (AMF) enhance plant resistance against various pathogens (Harrier and Watson, 2004; Pozo et al., 2005; Bi et al., 2007). Mycorrhiza colonization of onion (*Allium cepa*) by *Funneliformis mosseae* (syn. *Glomus mosseae*) significantly alleviated the pink root disease caused by *Pyrenochaeta terrestris* (Safir, 1968). The verticillium wilt was significantly reduced in cotton plants colonized by AMF, *F. mosseae*, *G. versiforme*, and *Sclerocystis sinuosa* (Liu, 1995). Mycorrhizal colonization improved tomato resistance to an array of diseases caused by *Erwinia carotovora* (García-Garrido and Ocampo, 1988), *Fusarium oxysporum* f. sp. *lycopersici* (Akköprü and Demir, 2005), *Phytophthora nicotianae* var. parasitica (Cordier et al., 1996), *P. parasitica* (Cordier et al., 1998), and *Pseudomonas syringae* (García-Garrido and Ocampom, 1989). Mycorrhizal symbiosis also enhanced tomato resistance to foliar disease of early blight (Fritz et al., 2006). Common mycorrhizal networks between tomato plants conferred protection of neighbors against early blight (Song et al., 2010). Use of AMF provides a sustainable alternative for crop disease management (Liu et al., 2007; Elsen et al., 2008). However, the underlying mechanism of AMF-induced disease resistance remains elusive. A significant transcriptional reprogramming occurs in host plant upon mycorrhizal colonization (López-Ráez et al., 2010; Jung et al., 2012). The induction of plant defenses during mycorrhization plays a vital role in mycorrhiza-enhanced resistance (Pozo and Azcón-Aguilar, 2007; Jung et al., 2012).

Colonization or infection by certain beneficial microbes or necrotizing pathogens provokes a specific physiological state in plants called “priming” (Hao et al., 2012; Aimé et al., 2013). The primed state in plants can also be induced by various natural and artificial compounds, such as β-aminobutyric acid (BABA), jasmonic acid (JA), and salicylic acid (SA) (Jakab et al., 2001; Worrall et al., 2012). The primed plants show quicker and/or stronger induction of various cellular defense responses following exposure to either pathogens herbivore insects, or abiotic stress (Kuc, 1987; Ton et al., 2006; Jung et al., 2009; Slaughter et al., 2012; Ye et al., 2013). Recent studies demonstrate that the defense priming in *Arabidopsis thaliana* plants can be transferred to their progeny, conferring better protection from pathogen attack (Slaughter et al., 2012). Tomato plants grown from seeds treated with JA and BABA displayed enhanced resistance against insect herbivory and powdery mildew disease (Worrall et al., 2012).

The objectives of this study are to investigate the effects of pre-inoculation of tomato plants with *Funneliformis mosseae* on resistance to early blight disease caused by *Alternaria solani*, as well as on defense responses in pre-inoculated and un-inoculated tomato plants. We hypothesized that AMF pre-colonization primes tomato plants and initiates host defense response upon subsequent pathogen attack. In general, SA signaling triggers resistance against biotrophic and hemibiotrophic pathogens, whereas the JA pathway activates resistance against necrotrophic pathogens (Glazebrook, 2005; Robert-Seilaniantz et al., 2011). Since the pathogen *A. solani* exhibits a necrotrophic lifestyle, we examined the role of the JA pathway in AMF-induced priming in tomato by using transgenic tomato plants that overexpress the prosystemin gene (*35S::PS*) and plants with a mutation in the JA biosynthetic pathway (*spr2*). In tomato plants, systemic induction of JA-dependent defense responses is mediated by an 18-amino-acid peptide signal called systemin (Howe and Ryan, 1999). Tomato transgenic line 35S::prosystemin (*35S::PS*) that overexpress prosystemin, the systemin precursor, exhibit constitutive expression of several JA-regulated defensive proteins including proteinase inhibitors and polyphenol oxidase (Chen et al., 2006).

## Materials and Methods

### Experimental Design

Tomato plants (*S. lycopersicum* Mill. cv. Jin Bao) were inoculated with mycorrhizal fungus *Funneliformis mosseae* (*syn. G. mosseae*) Gerdemann & Trappe BEG 167. *A. solani* Sorauer (ACCC36110) was inoculated to cause tomato early blight disease. Two tomato plants were cultivated in a rectangular pot (24 cm in length, 18 cm in height, 12 cm in width). To examine effects of mycorrhizal colonization on pathogen infection and tomato defense response, we designed four treatments (CK, As, Fm, and Fm+As): (1) CK: control plants without AMF and pathogen inoculation; (2) As: plants inoculated with *A*. *solani* only; (3) Fm: plants inoculated with *F. mosseae* only; (4) Fm+As: plants inoculated with *F. mosseae* and later challenged with *A*. *solani*. For mycorrhizal inoculation, the sand substrate (100 g) containing the inoculum of *F. mosseae* was applied to each plastic pot in treatments Fm and Fm+As before sowing. Leaves of tomato plants were harvested 18, 65, 100, and 140 h after pathogen inoculation for real-time RT-PCR and enzymatic analysis.

### Plant and Fungal Materials

Tomato seeds were sterilized with H_2_O_2_ (10%) for 10 min and rinsed five times with sterile distilled water. The seeds were then sowed in autoclaved sand-soil mixture (1:1).

The inocula of *F. mosseae* were propagated by using corn plants (*Zea mays* L.) cultured in autoclaved sand (Chellappan et al., 2002). A mixture of corn roots and rhizospheric sand from trap cultures containing approximate 35 AMF propagules per gram was used for AM inoculation.

The pathogen was cultured for 6 day on potato dextrose broth with 100 mg/l streptomycin sulfate, at 28°C in darkness and on a shaker at 150 rpm. Then the fungal culture was centrifuged at 1000 *g*, re-suspended in sterilized distilled water, and re-centrifuged. The concentration of AMF spores was measured and adjusted to 10^6^ conidia per milliliter using a hemacytometer.

To reveal the role of the jasmonate (JA) signaling pathway in mycorrhiza-induced systemic priming of disease resistance against *A. solani*, both overexpressing 35S::prosystemin and defective *spr2* mutant lines, as well as their corresponsive wild-type (WT) tomato plants, were used to compare their differential responses to *A. solani* infection after mycorrhizal colonization by AMF *F. mosseae.* Tomato cv Castlemart was used as the WT parent, the mutant line *suppressor of prosystemin-mediated responses2* (*spr2*) was derived from cv Castlemart (Li et al., 2003). The *35S::PS* transgenic plants were developed from the seeds collected from a 35S::prosys/35S::prosys homozygous line (Chen et al., 2006) that was backcrossed five times using the recurrent parent cv Castlemart.

### Bioassay

To determine mycorrhizal colonization on tomato disease resistance, a bioassay was carried out to compare the disease incidence and disease severity index (see definitions below) between non-mycorrhizal and mycorrhizal tomato plants. The brown loam soil collected from the campus of South China Agricultural University in Guangzhou (China) contained 1.52% organic matter, 0.789 g/kg total N, 0.42 g/kg total P, 1.76 g/kg total K, 35.93 mg/kg available N, 1.30 mg/kg available P, and 37.14 mg/kg available K with a pH of 4.68. The soil autoclaved at 121°C for 2 h was mixed with sterilized sand at a ratio of 2:1. The mixture was used as culture medium of tomato plants. The inocula (225 g) of AMF *F. mosseae* were incorporated into the obtained mixture (1.5 kg) for mycorrhizal inoculation. The same amount of mixture (1.5 kg) and sterilized sand (225 g) was applied to each non-mycorrhizal control pot. The control pots were watered with a soil filtrate obtained by shaking non-pasteurized rhizospheric sand with sterilized water then filtering it through a Watman No 1 filter, to exclude possible effect of other soil microorganisms. The filtrate contained the natural soil microbial population without AMF inocula.

Two pre-germinated tomato seeds were transplanted into each plastic pot with the growth substrate. Ten days later, the seedlings were thinned to one plant per pot. The plants were grown in a growth chamber at 25 ± 1°C with a 16 h photoperiod, 150 Md/m^2^/s PAR and 60% relative humidity. The seedlings were watered daily and fertilized every 5 days with 50 mL of Hoagland nutrient solution (5 ml 1 M KNO_3_, 5 ml 1 M Ca(NO_3_)_2_, 1 ml 1 M MgSO_4_, 2 ml 1 M KH_2_PO_4_, 1 ml 46 mM H_3_BO_3_, 1 ml 11 mM MnCl_2_, 1 ml 1 mM ZnSO_4_, 1 ml 3.5 mM CuSO_4_ and 1 ml 17.7 mM FeEDTA in one liter water). Thirty-five days after transplanting, tomato leaves in each pot were carefully sprayed with 30 ml of a conidia suspension (10^6^ conidia/ml) of *A*. *solani*. All plants were covered with an air-tight plastic bag during pathogen infection to maintain the high relative humidity facilitating spore germination.

The incidence and severity of tomato early blight were measured 10 d post pathogen inoculation. Disease incidence was indicated by percentage of diseased tomato leaves. Disease severity was estimated using a Disease Index (DI) calculated from disease grades 0–5 (Sriram et al., 1997), using the following formula:

(1) $$DI=\frac{Sumofindividual\times leafratings}{Maximumdiseasescore\times Numberofleavessample}\times \;100\;$$

Individual leaf ratings in the formula refer to disease grade of each leaf of tomato. The maximum disease score refers to the maximum disease grade observed during the entire period of the experiment. Fifty root samples (1 cm in length) were collected from each plant, cleaned and stained to examine AM colonization by the ink-vinegar staining method (Vierheilig et al., 1998; Mukerji et al., 2002).

### Enzyme Assays

The experiment setups for enzyme assays and real-time RT-PCR analysis were the same as those for disease bioassays. PAL activity was determined as the rate of the conversion of L-phenylalanine to trans-cinnamic acid at 290 nm. Leaf samples (0.2 g) were harvested from the different treatment conditions (CK, Fm, As, and Fm+As) and ground using liquid nitrogen and homogenized in 1 ml ice cold 0.05 M sulfate buffer, pH 8.8 containing 7 mM 2-mercaptoethanol and 0.1 g insoluble polyvinylpyrrolidone. The homogenate was centrifuged at 12000 *g* for 20 min. The supernatant was used for enzyme analysis. PAL activity was determined spectrophotometrically (Edwards and Kessmann, 1992).

Lipoxygenase (LOX) activity was measured as conjugated diene formation (Macri et al., 1994). Leaf samples (0.2 g) were ground using liquid nitrogen and extracted with 1 ml ice-cold 0.5 M TRIS-HCl buffer (pH 7.6) and centrifuged at 12 000 *g* for 15 min at 4°C. The supernatant was kept at 4°C until used. The substrate contained 1.6 mM linoleic acid and 0.5% (v/v) Tween 20 in 0.1 M phosphate buffer (pH 7.6). The reaction was initiated by the addition of 0.2 ml crude extract in 4.8 ml of the substrate. Diene formation was followed as increase of absorbance at 234 nm.

Leaf samples (0.1 g) were ground in liquid nitrogen and extracted with 2 ml 0.05 M sodium acetate buffer (pH 5.0) and centrifuged at 12 000 *g* for 15 min at 4°C. The supernatant was used for the enzyme assay of β-1,3-glucanase and chitinase. β-1,3-Glucanase activity was assayed by the laminarindinitrosalicylic acid method (Pan et al., 1991). The chitinase activity was analyzed as described (Boller and Mauch, 1988).

### Real-time RT-PCR Analysis

Differential expression of selected genes was verified by real time -polymerase chain reaction (RT-PCR) using the RNA samples isolated from tomato leaves obtained from the four treatments. The total RNA was extracted and isolated as described by Kiefer et al. (2000), with slight modification. Fresh leaves (0.2 g) were ground with a mortar and pestle in liquid nitrogen, and the powdered tissue transferred to a 2 ml Eppendorf tube, then 1000 μl TRIzol reagent (Invitrogen) was added and mixed. After incubation for 8–10 min on ice, 200 μl chloroform was added and mixed. Following 5 min incubation at room temperature, the mixture was centrifuged at 12,000 *g* for 15 min at 4°C. The supernatant was transferred to a 1.5 ml Eppendorf tube and 500 μl isoamylalcohol was added, followed by vortexing at room temperature for 10 min and centrifugation at 13,000 *g* for another 10 min at 4°C. The supernatant was discarded and the pellet was washed with 1 ml 75% ethanol (v/v), dissolved in 30 μl RNAse free water and kept at -80°C until used. RNA integrity was checked on a denaturing agarose gel electrophoresis; the concentration was determined spectrophotometrically before further use.

The expression patterns of defense-related genes (*PAL*, *LOX*, *AOC*, *PR1*, *PR2*, and *PR3*) in different treated tomato leaves were analyzed by using Real Time-PCR. The primers for target genes *PAL*, *LOX*, *AOC*, *PR1*, *PR2*, and *PR3* were designed by Primer 3.0 software (Applied Biosystems, http://fokker.wi.mit.edu/primer3/input.htm) based on tomato mRNA sequences deposited in GenBank. The gene-specific primer sequences used are listed in **Table 1**. *Ubi3* (Accession No. X58253) was used as a reference. Proteinase inhibitor II (*Pin2*) gene was chosen because it is typical jasmonic acid responsive gene systemically induced upon wounding (Farmer and Ryan, 1992). Real-time PCR reactions were carried out with 0.2 μl (0.15 μM) of each specific primers, 1 μl (50 ng) cDNA, and 12.5 μl of the SYBR green master mix (Quanti Tech SYBR Green kit, Qiagen, Gmbh Hilden, Germany), and the final volume was made up to 25 μl with RNase-free water. In the negative control, cDNA was replaced by RNase free water. The reactions were performed on a DNA Engine Opticon 2 Continuous Fluorescence Detection System (MJ Research Inc., Waltham, MA, US). The program used for real-time PCR was 3 min initial denaturation at 95°C, followed by 35 cycles of denaturation for 20 s at 95°C, annealing for 20 s (*PAL*: 57°C; *LOX*: 56.9°C; *AOC*: 56.5°C; *PR1*: 55.4°C; *PR2*: 56°C; *PR3*: 58°C; *Pin2:* 60.0°C; *Ubi3*: 58°C) and extension for 20 s at 72°C. The fluorescence signal was measured immediately after incubation for 2 s at 75°C following the extension step, which eliminates possible primer dimer detection. At the end of the cycles, melting temperatures of the PCR products was determined between 65 and 95°C. The specificity of amplicons was verified by melting curve analysis and agarose gel electrophoresis. Three independent biological replicates for each treatment were used for qRT-PCR analyses.

**Table 1:** Specific primer for real-time PCR.

Gene	Accession No.	Primer sequence (5′ to 3′)	PCR product size
*LeLOX*	U13681	F: 5′-ATCTCCCAAGTGAAACACCACA-3′ R: 5′-TCATAAACCCTGTCCCATTCTTC-3′	109 bp
*LeAOC*	AW624058	F: 5′-CTCGGAGATCTTGTCCCCTTT-3′ R: 5′-CTCCTTTCTTCTCTTCTTCGTGCT-3′	115 bp
*LePR1*	DQ159948	F: 5′-GCCAAGCTATAACTACGCTACCAAC-3′ R: 5′-GCAAGAAATGAACCACCATCC-3′	139 bp
*LePR2*	M80604	F: 5′-GGACACCCTTCCGCTACTCTT-3′ R: 5′-TGTTCCTGCCCCTCCTTTC-3′	81 bp
*LePR3*	Z15140	F: 5′-AACTATGGGCCATGTGGAAGA-3′ R: 5′-GGCTTTGGGGATTGAGGAG-3′	128 bp
*LePIN2*	X94946	F:5′-AATTATCCATCATGGCTGTTCAC-3′ R: 5′- CCTTTTTGGATCAGATTCTCCTT-3′	254 bp
*LePAL*	AW035278	F: 5′-CTGGGGAAGCTTTTCAGAATC-3′ R:5′-TGCTGCAAGTTACAAATCCAGAG-3′	150 bp
*LeUBI3*	X58253	F: 5′- TCCATCTCGTGCTCCGTCT -3′ R:5′-GAACCTTTCCAGTGTCATCAACC-3′	144 bp

### Statistical Analysis

For each treatment, three replicates were maintained in a completely randomized design. SAS 8.0 (SAS Institute, Cary, North Carolina) package for windows was used for statistical analysis. The data were analyzed with a one-way analysis of variance with the significant differences among means identified by Tukey’s multiple range test (*P* < 0.05).

## Results

### Induction of Disease resistance by Mycorrhizal Colonization

**FIGURE 1:**
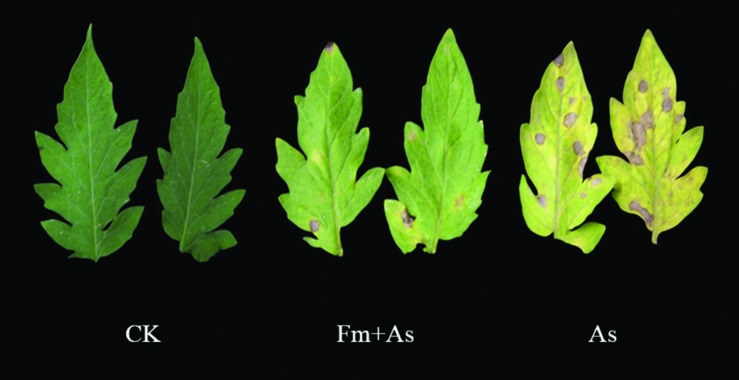
**Disease symptoms of early blight in leaves of tomato plants with or without mycorrhizal colonization by *Funneliformis mosseae.*** The photos were taken 10 days after pathogen inoculation by *Alternaria solani*. Three treatments included: (1) CK: control plants without pathogen and mycorrhizal inoculation; (2) As: plants inoculated with *A*. *solani* only; (3) Fm+As: plants inoculated with both *F. mosseae* and *A*. *solani*.

Inoculation of tomato plants with the AMF, *F. mosseae*, led to a significant decrease in disease incidence and disease severity of early blight compared to the control plants without mycorrhizal inoculation (**Table 2**). Disease incidences and indices were reduced in mycorrhizal plants by 54.3% and 72.8%, respectively, 10 d after pathogen inoculation. Mycorrhizal plants had significantly fewer disease symptoms than non-mycorrhizal plants (**Figure 1**). Furthermore, disease development in AMF-inoculated plants was significantly slower. Microscopic observation showed that the mycorrhizal infection rate was 55.1% in the inoculated plants (**Table 2**).

**Table 2:** Mycorrhizal colonization rates, disease incidences, and indices of tomato plants inoculated with either *Funneliformis mosseae*, *Alternaria solani*, or both.

Microbial inoculation	Disease incidence (%)	Disease index (%)	Mycorrhizal colonization (%)
Non-inoculation control *F. mosseae* *A. solani* *F. mosseae* and *A. solani*	0 c 0 c 63 ± 4.2 a 40.1 ± 5.3 b	0 c 0 c 44.5 ± 2.6 a 17.2 ± 0.8 b	0 c 60.3 ± 1.7 a 0 c 55.1 ± 1.5 b

*Four sets of bioassays were independently carried out and three pots per treatment were set up for each set of bioassays. Values are means ±*SE*. Significant differences (*P* < 0.05 using Tukey *post hoc* test) among treatments in the same column are indicated by different letters.*

### Induction of Defense-related Enzymes by Mycorrhizal Colonization

**FIGURE 2:**
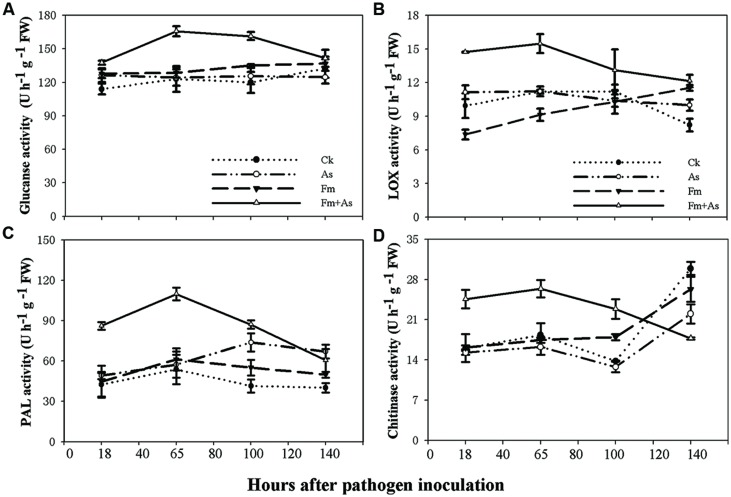
**Activity levels of defense-related enzymes in tomato leaves in response to mycorrhizal colonization and pathogen infection.** The tomatoes were pre-inoculated with mycorrhizal fungus *Funneliformis mosseae* and later inoculated with *A. solani*, the causal agent of early blight disease of tomato. Four defense-related enzymes are β-1,3-glucanase **(A)**, lipoxygenase (LOX) **(B)**, phenylalanine ammonia-lyase (PAL) **(C)**, and chitinase **(D)**. Four treatments included: (1) CK: control plants without pathogen and mycorrhizal inoculation; (2) As: plants inoculated with *A*. *solani* only; (3) Fm: plants inoculated with *F. mosseae* only; (4) Fm+As: plants inoculated with both *F. mosseae* and *A*. *solani*. Values are means ± SE from three sets of independent experiments with three pots per treatment for each set of experiments. Significant differences among treatments were tested at *P* = 0.05 by Tukey *post hoc* test.

To examine effects of mycorrhizal colonization on defense responses in host plants in presence of pathogen infection tomato plants were subjected to four treatments: (1) CK: control without fungal inoculation; (2) As: inoculation with *A*. *solani*; (3) Fm: inoculation with *F. mosseae*; (4) Fm+As: inoculation with both *F. mosseae and A*. *solani*. Four defense-related enzymes, including PAL, LOX, chitinase, and β-1,3-glucanase were analyzed in the leaves of tomato plants. Mycorrhizal pre-inoculation significantly enhanced activities of the four enzymes in the leaves upon pathogen infection (**Figures 2A–D**). The activities of all tested enzymes were significantly higher in treatment Fm+As after the pathogen inoculation and reached a maximum at 65 h. The activity of β-1,3-glucanase was increased by 34.7, 33.3, and 28.8%, respectively, relative to those in treatments CK, As, and Fm 65 h post the pathogen inoculation (**Figure 2A**). However, the activity of β-1,3-glucanase did not differ significantly between the other treatments (CK, As, and Fm) (**Figure 2A**).

The enzymatic activity of LOX in treatment Fm+As was significantly higher after pathogen inoculation (**Figure 2B**). LOX activity in treatment Fm+As increased by 48.1, 32.2, and 99.5% at 18 h after pathogen inoculation compared to treatments CK, As, and Fm, respectively, and increased by 38.1, 37.8, and 68.1% at 65 h after pathogen inoculation, respectively.

Although mycorrhization led to some increase in PAL activity in treatment Fm, the PAL induction was more pronounced in treatment Fm+As. The PAL activity in treatment Fm+As was, on average, higher by 104.3, 74.9, and 79.5% than that of treatment CK, As, and Fm, respectively, at 65 h after pathogen inoculation (**Figure 2C**). In contrast, the difference in PAL activity among treatments CK, As, and Fm were less variable. In particular, the PAL activity was not significantly different among treatment CK, As, and Fm at 18 and 65 h following pathogen inoculation.

Chitinase activity in mycorrhizal pre-inoculated plants (Fm+As) was significantly higher at 18, 65, and 100 h after the pathogen inoculation (**Figure 2D**). It displayed increases of 44.1, 62.1, and 55.1% in treatment Fm+As compared to treatments CK, As, and Fm, respectively, at 65 h after pathogen inoculation.

### Transcript Induction of Defense-related Genes by Mycorrhizal Colonization

**FIGURE 3:**
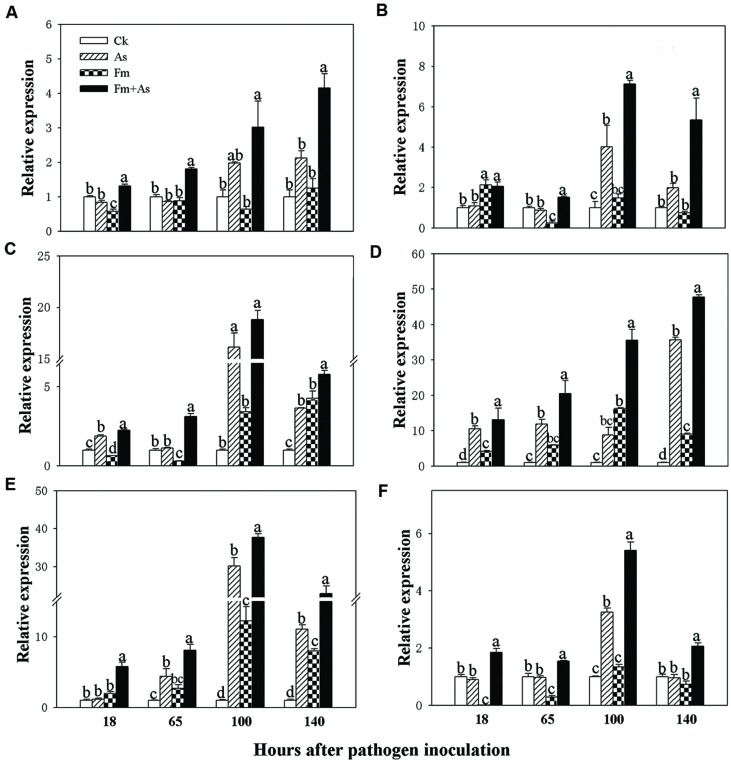
**Transcripts of defense-related genes in tomato leaves in response to mycorrhizal colonization and pathogen infection.** The tomates were pre-inoculated with mycorrhizal fungus *Funneliformis mosseae* and later inoculated with *A. solani*, the causal agent of early blight disease of tomato. Quantitative real time RT-PCR was used to detect the transcripts of six defense-related genes encoding *PAL* **(A)**, *LOX* **(B)**, allene oxide cyclase (*AOC*) **(C)**, pathogen-related proteins (*PR1*) **(D)**, β-1,3-glucanase (basic type *PR-2*) **(E)**, and chitinase (*PR-3*) **(F)**. Four treatments included: (1) CK: control plants without pathogen and mycorrhizal inoculation; (2) As: plants inoculated with *A*. *solani* only; (3) Fm: plants inoculated with *F. mosseae* only; (4) Fm+As: plants inoculated with both *F. mosseae* and *A*. *solani*. Values are means ± SE from three sets of independent experiments with three pots per treatment for each set of experiments. Significant differences (*P* < 0.05 using Tukey *post hoc* test) among treatments in a group are indicated by different letters above bars.

To determine whether mycorrhizal colonization enhances the disease resistance and defense response by inducing transcription of defense-related genes (Pozo et al., 2005), the expression patterns of the six genes (*PAL*, *LOX*, *AOC* (encoding allene oxide cyclase for JA biosynthesis), *PR1*, *PR2*, and *PR3*) were analyzed by using real-time RT-PCR from tomato leaves 18, 65, 100, and 140 h post pathogen inoculation. Mycorrhizal pre-inoculation of tomato plants with *F. mosseae* and later pathogen inoculation with *A*. *solani* (treatment Fm+As) induced accumulation of *PAL*, *LOX*, *AOC*, *PR1*, *PR2*, and *PR3* transcripts over basal levels present in the leaves of un- inoculated control (CK), sole *A*. *solani* inoculation (As), and *F. mosseae* colonization (Fm) treatments 18, 65, 100, and 140 h after pathogen inoculation (**Figure 3**). The expression levels of *PAL, LOX*, and *AOC* were induced approximately 3.0, 7.1, and 18.8-fold at 100 h, and by 4.1, 5.3, and 5.8-fold at 140 h post pathogen inoculation, respectively, in response to dual inoculation with the AMF and the pathogen (Fm+As) relative to the non-mycorrhizal control (CK) (**Figures 3A–C**). Mycorrhizal pre-colonization induced transcripts of *PR1*, *PR2*, and *PR3* by 20.4, 35.5, and 47.7-fold at 65 h, by 8.0, 37.7, and 22.9-fold at 100 h, respectively (CK) (**Figures 3D–F**). Pathogen infection alone (treatment As) induced transcripts of the six genes in the leaves of non-preinoculated tomato plants, but the induction was much less and slower compared with that in mycorrhizal and pathogen-infected plants (treatment Fm+As). Mycorrhizal colonization (treatment Fm) alone did not induce gene expression of *PAL* and *PR3* (**Figures 3A,F**). Although sole mycorrhization up-regulated transcripts of *PR1*, *PR2*, and AOC at 100 and 140 h, the induction was even weaker than that by pathogen infection.

### Role of Jasmonate Signaling Pathway in AMF-induced Disease Resistance

**FIGURE 4:**
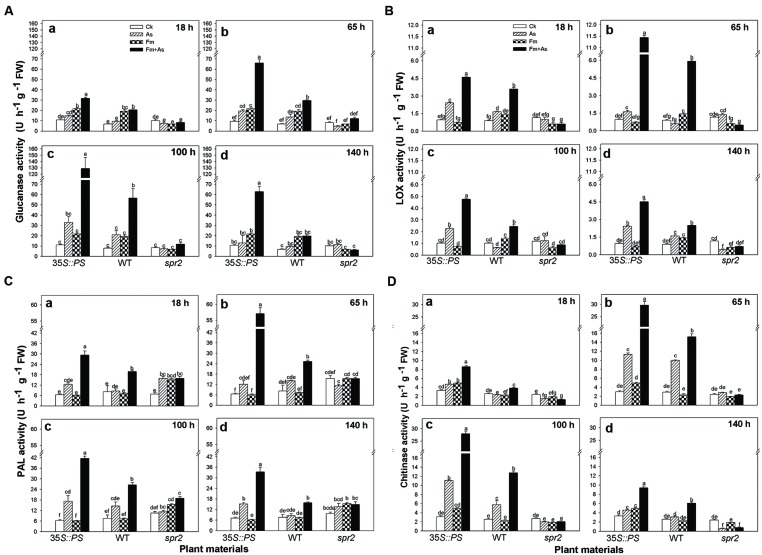
**Levels of defense-related enzymes in leaves of tomatoes with mycorrhizal colonization and pathogen infection.** Wild-type (WT) and mutant plants (*35S::PS* and *spr2*) of tomato were pre-inoculated with mycorrhizal fungus *Funneliformis mosseae* and later inoculated with *A. solani*, the causal agent of early blight disease of tomato. Four defense-related enzymes are β-1,3-glucanase **(A)**, LOX **(B)**, PAL **(C)**, and chitinase **(D)**. Enzymatic activities were analyzed 18 (a), 65 (b), 100 (c) and 140 h (d) after pathogen inoculation. Four treatments included: (1) CK: control plants without pathogen and mycorrhizal inoculation; (2) As: plants inoculated with *A*. *solani* only; (3) Fm: plants inoculated with *F. mosseae* only; (4) Fm+As: plants inoculated with both *F. mosseae* and *A*. *solani*. Three tomato genotypes included: (1) WT: wild type plant; (2) *35S::PS*: Prosystemin-overexpressing *35S::PS* plant; (3) *spr2*: JA biosynthesis mutant plant. Values are means ± SE from three sets of independent experiments with three pots per treatment for each set of experiments. Significant differences among treatments were tested at *P* = 0.05 by Tukey *post hoc* test.

Three tomato genotypes: a WT plant, a JA biosynthesis mutant (*spr2*), and a prosystemin-overexpressing *35S::PS* plant, were used to identify the role of the JA signaling pathway in mycorrhiza-induced disease resistance against *A*. *solani*. The plants of the three genotypes were subjected to the same four treatments as above. There was no significant difference in activities of the four defense-related enzymes in control plants of the three genotypes (**Figures 4A–D**). However, the three genotypes showed large differences in activities of the four defense-related enzymes in response to pathogen infection (As) and dual inoculation with the pathogen and mycorrhizal fungus (Fm+As). The *35S::PS* plants showed higher induction of enzymatic activities in *A*. *solani*-inoculated plants compared to the other two genotypes. Most importantly, mycorrhizal pre-inoculated *35S::PS* plants showed the highest induction of enzymatic activities (**Figures 4A–D**). β-1,3-Glucanase activity in treatment Fm+As was increased by 1083.7, 291.3, and 495.5% at 100 h post pathogen inoculation compared with that in treatments CK, As, and Fm, respectively (**Figure 4Ac**). Similarly, chitinase activity in treatment Fm+As was increased by 795.2, 161.5, and 498.8% at 65 h compared with the other three treatments (**Figure 4Db**). Similar trends were observed for LOX and PAL activities (**Figures 4B,C**). The LOX and PAL activities were increased in mycorrhizal pre-inoculated *35S::PS* and WT plants after pathogen inoculation, but *35S::PS* plants exhibited significantly higher LOX and PAL activities as compared to WT plants (**Figures 4B,C**). The *35S::PS* plants showed 48.5, 125.0, 56.0, and 111.4% higher PAL activity in treatment Fm+As than that of WT plants 18, 65, 100, and 140 h after pathogen inoculation, respectively (**Figure 4Ca–d**). In contrast, the four tested enzymes were not induced in the *spr2* plants in response to pathogen inoculation (As) and dual inoculation (Fm+As) (**Figures 4A–D**).

**FIGURE 5:**
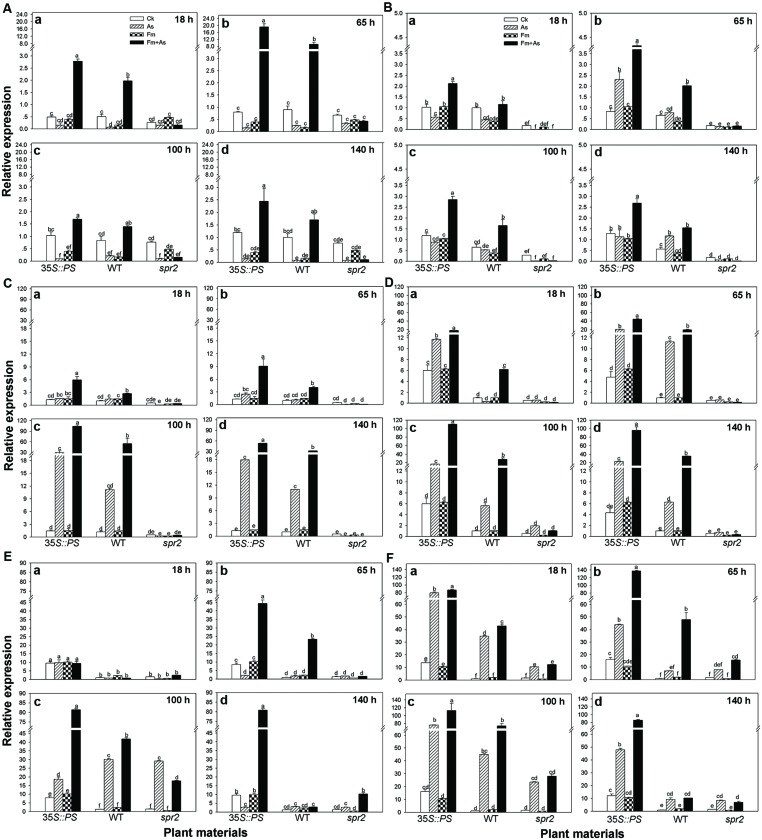
**Transcripts of defense-related genes in leaves of tomatoes with mycorrhizal colonization and pathogen infection.** WT and mutant plants (*35S::PS* and *spr2*) of tomato were pre-inoculated with mycorrhizal fungus *Funneliformis mosseae* and later inoculated with *A. solani*, the causal agent of early blight disease of tomato. Quantitative real time RT-PCR was used to detect the transcripts of six defense-related genes encoding the *PAL* **(A)**, *LOX* **(B)**, (*Pin2*) **(C)**, pathogen-related proteins (*PR1*) **(D)**, β-1,3-glucanase (basic type *PR-2*) **(E)**, and chitinase (*PR-3*) **(F)**. Transcript levels were quantified 18 (a), 65 (b), 100 (c), and 140 h (d) after pathogen inoculation. Four treatments included: (1) CK: control plants without pathogen and mycorrhizal inoculation; (2) As: plants inoculated with *A*. *solani* only; (3) Fm: plants inoculated with *F. mosseae* only; (4) Fm+As: plants inoculated with both *F. mosseae* and *A*. *solani*. Three tomato genotypes included: (1) WT: wild type plant; (2) *35S::PS*: Prosystemin-overexpressing *35S::PS* plant; (3) *spr2*: JA biosynthesis mutant plant. Values are means ± SE from three sets of independent experiments with three pots per treatment for each set of experiments. Significant differences among treatments were tested at *P* = 0.05 by Tukey *post hoc* test.

Mycorrhizal pre-inoculation on *35S::PS* and WT tomato plants resulted in strong induction of transcripts of defense-related genes (*PAL*, *LOX*, *Pin2*, *PR1*, *PR2*, and *PR3*) upon pathogen attack (treatment Fm+As) (**Figures 5A–F**). The highest induction of the six defense-related genes was found in the mycorrhizal *35S::PS* plants. While no induction of *PAL* expression was found with pathogen inoculation alone (As) or AMF inoculation alone (Fm), dual inoculation with the AMF, and pathogen induced *PAL* 24.0- and 10.2-fold in *35S::PS* and WT plants, respectively, 65 h after pathogen inoculation (**Figure 5Ab**). Similarly, AMF pre-inoculation and later pathogen infection induced *PR1* 18.3-, 6.5-, and 17.5-fold in *35S::PS* plants as compared to that in treatments CK, As, and Fm, respectively, 100 h after pathogen inoculation (**Figure 5Dc**). Although pathogen infection alone induced *PR1* transcripts in *35S::PS* and WT plants, the induction was significantly lower relative to treatment Fm+As (**Figure 5D**). No induction was found in the *spr2* plants in response to pathogen inoculation (As) or dual inoculation (Fm+As) (**Figures 5A–F**). Similar inductions of *PR3* and *Pin2* transcripts were observed in *35S::PS* and WT plants, but there was no induction in the JA-deficient *spr2* mutant (**Figures 5C,F**).

Bioassays showed that mycorrhizal pre-inoculation on *35S::PS* and WT tomato plants significantly reduced disease incidence and disease severity of early blight relative to AMF un-inoculated control plants (**Table 3**). Mycorrhizal colonization led to 17.6 and 15.6% reductions in the disease incidence and disease severity, respectively, in WT plants, and 19.7 and 20.4% reductions, respectively, in *35S::PS* plants. In contrast, mycorrhizal inoculation did not affect the disease incidence and severity of the *spr2* mutant plants. On the other hand, the *spr2* plants had the lowest mycorrhizal colonization rate and the highest disease incidence and severity among the three tomato genotypes (**Table 3**).

**Table 3:** Mycorrhizal colonization rates, disease incidences, and indices of AMF-inoculated and un-inoculated tomato plants inoculated by AMF *Funneliformis mosseae* and pathogen *A. solani.*

Microbial inoculation	Tomato genotype	Disease incidence (%)	Disease index (%)	Mycorrhizal colonization (%)
*F. mosseae* and *A. solani*	WT	60.2 ± 1.3 cd	36.8 ± 1.3 c	40.6 ± 4.1 a
	*35S::PS*	54.5 ± 4.4 d	28.5 ± 1.7 d	41.2 ± 1.2 a
	*spr2*	86.4 ± 2.1 a	53.4 ± 1.4 a	14.0 ± 1.9 b
*A. solani*	WT	73.1 ± 3.1 b	43.6 ± 2.4 b	0 c
	*35S::PS*	67.9 ± 3.6 bc	35.8 ± 1.2 c	0 c
	*spr2*	89.6 ± 1.4 a	56.2 ± 1.4 a	0 c

*Three tomato genotypes included: (1) WT: wild type plant; (2) *35S::PS*: Prosystemin-overexpressing *35S::PS* plant; (3) *spr2*: JA biosynthesis mutant plant. Four sets of bioassays were independently carried out and three pots per treatment were set up for each set of bioassays. Values are means ± SE. Significant differences (*P* < 0.05 using Tukey *post hoc* test) among treatments in the same column are indicated by different letters.*

## Discussion

In last two decades, early blight has become a major disease of tomato in many parts of China (Dong et al., 2015). This study shows that tomato early blight can be alleviated through mycorrhizal inoculation, which is consistent with previous finding by Fritz et al. (2006). The enhanced disease resistance was not due to improved phosphorus nutrient, though the underlying mechanism was not clear (Fritz et al., 2006). Mycorrhizal fungi are ideal biocontrol agents because they are natural soil-borne biota and can establish stable and long lasting mutualistic symbiosis with the roots of most vascular plant species, including most crops (Smith and Read, 2008). Mycorrhizal associations benefit not only plant nutrient absorption (Smith and Read, 2008), but also plant resistance to diverse abiotic stresses (Ruiz-Lozano et al., 1996) and soil-borne fungal pathogens (Harrier and Watson, 2004; Bi et al., 2007). More interestingly, AM symbiosis also enhances plant resistance against foliar pathogens such as fungal pathogens [e.g., *Botrytis cinerea* (Pozo et al., 2010; Fiorilli et al., 2011) and *A. solani* (Fritz et al., 2006)], bacteria [e.g., *Xamantomonas campestris* (Liu et al., 2007) and viruses (e.g., *Tomato yellow leaf curl Sardinia virus* (Maffei et al., 2014)].

Induction of pathogenesis-related (PR) proteins is believed an indicator of plant induced defense responses. Accumulation of chitinase and β-1,3-glucanase has been associated previously with induced systemic resistance in tomato to *A*. *solani* (Lawrence et al., 1996) and *Fusarium oxysporum* (Pozo et al., 2002). Basic isozymes of chitinase and β-1,3-glucanase inhibit *A*. *solani in vitro* (Lawrence et al., 1996). Early blight-resistant tomato lines possess higher levels of chitinase and β-1,3-glucanase than susceptible genotypes (Lawrence et al., 2000). *PR* genes have been frequently used as marker genes for systemic acquired resistance in many plant species (Mitsuhara et al., 2008). Our study showed that mycorrhizal pre-inoculation in tomato roots systemically induced both enzyme activities of chitinase and β-1,3-glucanase, and transcripts of the genes *PR1*, *PR2*, and *PR3* encoding PR proteins in the leaves of tomato. Pozo et al. (2002) found that *F. mosseae* colonization in tomato plants reduced both local and systemic disease symptoms caused by *Phytophthora parasitica* infection, as well as provoked local and systemic induction of chitinase, β-1,3-glucanase and superoxide dismutase.

Our study showed that mycorrhizal inoculation itself did not affect most enzyme activities and only slightly induced transcripts of *AOC, PR1*, and *PR2*. However, upon pathogen attack AMF pre-inoculation strongly induced defense responses of all six tested genes and four defense-related enzymes in tomato plants. Based on the results that plants inoculated with both *F. mosseae* and *A*. *solani* had less disease damage, higher levels of defense-related enzymatic activities and gene expression than the controls, or pathogen or mycorrhizal inoculations alone, we suggest that mycorrhizal colonization on tomato can prime plant defense responses against early blight disease.

Most studies on defense priming focus on priming signals triggered by herbivore-induced volatile compounds (Ton et al., 2006; Heil and Silva Bueno, 2007; Ramadan et al., 2011). Some studies show that priming of plant defense can also be triggered by certain beneficial micro-organisms (Pozo et al., 2005; van Hulten et al., 2006; van Wees et al., 2008), including AMF (Pozo and Azcón-Aguilar, 2007; Pozo et al., 2009; Jung et al., 2012). Mycorrhizal pre-inoculation results in significantly higher production of PR-1a and basic β-1,3-glucanases in tomato plants upon *Phytophthora* infection (Cordier et al., 1998; Pozo et al., 1999; Maldonado-Bonilla et al., 2008). RNA-seq-based transcriptome analysis showed that mycorrhization led to the transcriptional changes in categories of signaling, hormone metabolism, biotic, and abiotic stresses, and several differentially expressed genes were related to systemic defense priming (Cervantes-Gámez et al., 2015). Our study confirms that priming is an important mechanism operating in mycorrhiza-induced disease resistance.

Plant disease resistance is tightly manipulated through an interconnected network of signaling pathways of JA and SA. PAL is the key enzyme involved in the biosynthesis of the signal molecule, SA (Mauch-mani and Slusarenko, 1996). SA accumulates in cells undergoing hypersensitive response and it is essential for local and systemic resistance response (Gaffney et al., 1993; Makandar et al., 2012). Induction of PAL activity is a reliable indicator of plant resistance expression (Mauch-mani and Slusarenko, 1996). An increase in of PAL activity indicates that, upon pathogen attack, mycorrhizal colonization initiates SA signaling pathways and increase accumulation of phenolic compounds.

The JA signaling pathway has been demonstrated to play a vital role in mediating plant defense responses to chewing herbivore insects (Howe and Jander, 2008; Bosch et al., 2014) and necrotrophic pathogens (Glazebrook, 2005; Robert-Seilaniantz et al., 2011). External application of methyl JA primes *Arabidopsis* defense against caterpillar herbivory (Rasmann et al., 2012). Since *A. solani* is a necrotrophic pathogen, we examined the roles of the JA pathway in AMF-induced priming in tomato. LOX, AOC, and AOS (allene oxide synthase) are three important enzymes in JA biosynthesis (Hause et al., 2002; Schaller et al., 2005). Stronger and quicker induction of *LOX* and *AOC* in mycorrhizal plants suggest that mycorrhizal colonization can also provoke the JA pathway, which thereby increases broad-spectrum disease resistance (De Vos et al., 2005).

Use of JA biosynthesis (*spr2*) mutant and prosystemin-overexpressing *35S::PS* plants revealed that the JA signaling pathway mediated AMF-primed defense in tomato plants. Mycorrhizal *35S::PS* plants had significantly higher levels of defense-related enzyme activity and gene expression than mycorrhizal WT plants and non-mycorrhizal *35S::PS* plants in response to *A. solani* infection (**Figures 4** and **5**). Although pathogenic infection alone induced enzymatic activities and gene transcripts in WT plants, the induction was lower than that of pathogen-infected mycorrhizal plants. However, AMF pre-inoculation and pathogenic infection did not lead to induction of defense-related enzymes and genes in *spr2* plants. The mycorrhizal *35S::PS* plants were the most resistant to early blight and mycorrhizal *spr2* plants were the most susceptible (**Table 3**). Non-mycorrhizal *35S::PS* plants showed similar level of disease resistance to mycorrhizal WT plants. These results suggest that the JA pathway is required for AMF-induced systemic priming of defense against *A. solani.* Rasmann et al. (2012) showed that herbivory in the previous generation primed *Arabidopsis* and tomato for enhanced insect resistance, and *Arabidopsis* mutants that were deficient in JA perception did not exhibit inherited resistance, demonstrating that the JA pathway is required in mother plants for priming resistance in the next generation. Low mycorrhizal colonization rate (**Table 3**) may suggest that JA signalings are necessary for establishment of mycorrhizal association.

## Conclusion

Pre-inoculation of tomato plants with *F. mosseae* enhanced tomato resistance to early blight. Root colonization by AMF systematically induced the defense-related enzymes and genes in the leaves of tomato upon pathogen challenge. Our results suggest that mycorrhizal-induced disease resistance in tomato is associated with priming for an efficient activation of defense responses upon pathogen attack. The AMF-induced primed responses were systemic and the JA pathway is required for such responses. Since most land plants have symbiotic association with mycorrhizal fungi (Brundrett, 2002), use of mycorrhizal fungi as defense priming elicitors may be an important evolutionary strategy for plant defense against pathogens and it may serve as an important alternative for management of crop disease in sustainable agriculture.

## Conflict of Interest Statement

The authors declare that the research was conducted in the absence of any commercial or financial relationships that could be construed as a potential conflict of interest.
